# Reducing cheap talk? How monetary incentives affect the accuracy of metamemory judgments

**DOI:** 10.3758/s13421-024-01679-5

**Published:** 2025-01-23

**Authors:** Arndt Bröder, Sofia Navarro-Báez, Monika Undorf

**Affiliations:** 1 School of Social Sciences, University of Mannheim, L 13, 17, 68131 Mannheim, Germany; 2 Department of Psychology, Technical University of Darmstadt, Darmstadt, Germany

**Keywords:** Metamemory, Judgment accuracy, Monetary incentives

## Abstract

The accuracy of metacognitive judgments is rarely incentivized in experiments; hence, it depends on the participants' willingness to invest cognitive resources and respond truthfully. According to arguments promoted in economic research that performance cannot reach its full potential without proper motivation, metacognitive abilities might therefore have been underestimated. In two experiments (*N* = 128 and *N* = 129), we explored the impact of incentives on the accuracy of judgments of learning (JOLs), memory performance, and cue use in free recall of word lists. We introduced a payoff scheme with 5 cents maximum per judgment to promote the accuracy of predicting recall success while simultaneously discouraging strategic responding in the memory test. Incentivizing JOLs had no effect on memory performance. Metacognitive accuracy in terms of resolution (Kruskal's Gamma) was slightly improved in Experiment 1, but not in Experiment 2. On the more negative side, the incentives boosted JOLs indiscriminately, producing substantial overconfidence. A deeper analysis including cues like word concreteness, imagery, arousal, frequency, subjective relevance, and font size showed the usual and simultaneous cue effects on JOLs. However, cue effects were largely unaffected in size by incentivizing JOLs. In summary, incentives for accuracy do not improve the resolution of JOLs to an extent that outweighs the large inflation of overconfidence. Based on the current results, one cannot recommend the future use of incentivized studies in the field of metamemory.

## Introduction

The term *metacognition* encompasses knowledge and beliefs people have about their cognitive processes. For example, in a typical metamemory study using *judgments of learning* (JOLs), people predict whether they will later recall (or recognize) to-be-studied items. Other studies use *confidence judgments* (CJs), in which people postdict their performance after reporting answers in cognitive tasks. Generally, metacognition research relies on all kinds of different judgments that measure people’s assessments of their cognitive processes, and the accuracy of the judgments has been the target of much research (Halamish & Undorf, 2021; Koriat, 2007; Rhodes, 2016). For example, people show positive Kruskal-Gamma correlations between their JOLs and memory performance, thereby demonstrating some metacognitive abilities, but the correlations are far from perfect (see Bröder & Undorf, 2019). This might at first sight hint at limited abilities, but another interpretation is that many people may not show their full potential since the accuracy of judgments is typically not incentivized. Hence, JOLs might underestimate true metacognitive skills because people are simply not motivated to express their actual beliefs or invest more cognitive effort to derive more accurate beliefs. Probability theorists interested in the valid assessment of subjective probabilities laid the foundations for incentivization and betting methods "according to the basic idea that the resulting device should oblige each participant to express his true feelings" (de Finetti, 1962, p. 359). Based on this reasoning, it is widely believed in experimental economics that incentives motivate participants to be (a) more accurate and (b) more honest. In consciousness research, payoff schemes have been used to achieve unbiased reports of people's awareness or unawareness of stimuli, called “post-decision wagering” (Koch & Preuschoff, 2007; Persaud et al., 2007). Charness et al. (2021) discuss various incentive schemes widely used in experimental economics to achieve this goal.

In a comparison of the typical research traditions in experimental economics and psychology, Hertwig and Ortmann (2001) identify *monetary incentives* as an important difference. Whereas economists distrust the “cheap talk” of unincentivized judgments, psychologists tend to take the judgments at face value and assume that participants are both willing and able to respond truthfully. Hertwig and Ortmann point out several reasons why economists rather incentivize judgments with money: First, based on participants' self-interest and the provision of a clear optimization criterion, monetary incentives should elicit maximal performance and truthfulness. Second, financial incentives are easier to implement than other incentives, and there are typically no satiation effects for money (i.e., more is always better). Third, empirically, reduced response variability with financial incentives points to a higher degree of optimization.

In metacognition, incentives for judgment accuracy have been rarely investigated, yet, and for JOLs, such research is almost absent (we will discuss the few exceptions below). Hence, the aim of the current work was to evaluate whether proper monetary incentivization would improve the accuracy of JOLs. The remainder of this article is structured as follows: First, we look at general incentive effects on cognitive tasks. Second, we focus on metacognitive judgments where most studies have looked at postdictive confidence and only a few at prospective judgments, such as JOLs. Third, we discuss the methodological challenges in installing an appropriate incentive scheme for JOLs and present our solution. Finally, we present two experimental studies and discuss their results. To foreshadow these, we found inconsistent results concerning resolution with only a small, if any, positive effect of incentives on resolution in Experiment 1. On the other hand, there was a large detrimental effect of incentives on calibration in both Experiment 1 and Experiment 2, producing marked overconfidence. This mirrors effects previously reported for confidence judgments.

### Monetary incentives and accuracy in cognition

To explore how incentives affect cognition, Hertwig and Ortmann (2001) reviewed ten studies from the *Journal of Behavioral Decision Making* in which monetary incentive conditions were directly compared to unincentivized conditions. The authors conclude that “in the majority of cases where payments made a difference, they improved people’s performance” (p. 394), such as reducing framing effects or bringing bids closer to optimum. This seems to imply that without incentives, participants fall short of their potential. However, a series of more systematic reviews and meta-analyses are more ambiguous: Camerer and Hogarth (1999) reported a qualitative review of 74 studies with varying amounts of incentives for various judgment and decision tasks, questioning the self-interest and cost–benefit logic of experimental economics. They conclude that incentives are helpful only in tasks in which motivation increases effort, and, in turn, effort is helpful for the task at hand. They even point to a handful of studies in which incentives decreased performance, presumably because more effortful but less accurate strategies were used. This concurs with a systematic review of 131 experiments by Bonner et al. (2000), who conclude that “consistent with our hypothesis regarding task type, judgment and choice tasks are less likely than vigilance and detection tasks, memory tasks, and production and clerical tasks to have positive incentive effects” (p. 31). In line with this, Jenkins et al.’s (1998) meta-analysis found an effect of incentives on quantitative performance, but not on qualitative performance in various tasks, with the smallest effects in laboratory settings.

Since these reviews date back some time, we searched for newer studies that included the manipulation of incentives using an objective performance criterion. Positive effects have been reported occasionally: Mohd-Zanussi and Mohd-Ikandar (2007) found a positive effect on accuracy in accounting tasks. However, this effect was restricted to the tasks of low complexity whereas very difficult tasks were unaffected. Neumann et al. (2022) had participants predict the performance of university applicants in their first year. Here, incentivized participants deviated less from an efficient decision rule. Lawson et al. (2020) examined six classic biases from judgment and decision-making research (e.g., the conjunction fallacy or the default bias) and found only a 1.7 percentage points increase in accuracy with incentives as compared to the control group. Enke et al. (2023) used very high incentives in one condition that roughly matched the monthly income of their Kenyan participants to test the robustness of four cognitive biases (e.g., base-rate neglect, anchoring, cognitive reflection test, Wason’s selection task (WST)) and found more effort (as measured by response times), but only marginal accuracy increases. For the base-rate task and the WST, the improvements were restricted to the easy versions of the tasks. Finally, there are also studies with negative effects of incentives. For example, Meloy et al. (2006) found that incentives increased the distortion of information evaluation, and Krawczyk (2012) reports an even larger “better-than-average” bias when people were incentivized.

To summarize, the plausible notion that monetary incentives increase motivation and therefore performance has received at best mixed support. In particular, performance can only be increased in tasks where additional effort pays off in terms of accuracy. Since this may or may not be the case for JOLs, the question of whether monetary incentives can improve them is still open.

### Monetary incentives and metacognition

The accuracy of metamemory judgments like JOLs and CJs can be assessed with respect to two criteria: If judgments are expressed on percentage scales, the *calibration* is the overall numerical match between the average judgment and actual performance measured in percent remembered. The *resolution* (or sensitivity), on the other hand, is the ability of a person to distinguish the successful items from the unsuccessful ones (i.e., remembered items receive high judgments while forgotten items receive low judgments). There is a debate on how to measure resolution best (Bröder & Undorf, 2019; Masson & Rotello, 2009; Murayama et al., 2014; Spellman et al., 2008), but an often-used measure is the within-participant Kruskal-Gamma correlation across items, which we also refer to in this paper (Nelson, 1984). We will discuss some incentive studies for CJs and JOLs in turn.

The notion of *overconfidence* has been established in the literature for too much predictive or postdictive certainty about one’s performance (see Hoffrage, 2017, for an overview). When using percentage scales to express judgments (CJs or JOLs), overconfidence is operationalized as a positive difference between the mean judgment and the percentage correct. One method to elicit confidence judgments is to ask participants in numerical estimation to provide 90% confidence intervals (CIs) for the to-be-estimated values. If confidence were well calibrated, one would expect 90% of the correct values to be covered by the subjective intervals. However, the CIs are typically too narrow, thus producing fewer of the actual values being included. Meloy et al., (2006, Study 3) incentivized CI accuracy in one of two groups, and found a dramatically *lowered* rate of actual values covered (i.e., much larger overconfidence and narrower CIs). This increase in overconfidence was mediated by an increase in positive mood induced by the incentives.

A more common way of eliciting confidence judgments is the use of confidence rating scales. With this method, Lebreton et al., (2018, 2019) found in several experiments that positive rewards for accurate confidence judgments in a perceptual discrimination task or a simple reinforcement learning task increased confidence without increasing accuracy in the primary task. In consequence, the overconfidence that was already present in a neutral condition was intensified through the possibility of monetary wins, and it increased with the amount of the reward. When using a loss frame with potential penalties for inaccuracy rather than gains for accuracy, however, overconfidence was somewhat reduced. Hence, calibration was worse with positive incentives. In contrast, the resolution of confidence judgments was slightly increased by incentives, but this effect was not replicated by Hoven et al. (2022) using the same paradigm. A similar increase in overconfidence had been reported earlier by Yates et al. (1997) for US students answering trivia questions. Here, direct probability judgments were compared within subjects to probabilities inferred from wagering decisions involving minimal selling prizes. The same manipulation did not affect the calibration of Taiwanese students who were even more overconfident than the US participants.

Roch et al. (2011) had participants judge various aspects of teaching quality for videotaped teachers as well as metacognitive assessments of their own accuracy. Here again, incentives boosted confidence but did not impact accuracy or did so only to a lesser degree. Krawczyk (2012) investigated whether monetary incentives can reduce the “better-than-average” effect in which most people claim to be better than average. Counter to expectations, incentives aggravated this bias rather than reducing it, again pointing to increased overconfidence.

In sum, monetary incentives tend to boost confidence without an accompanying increase in the accuracy of the primary task. Two exceptions are the studies by Callender et al. (2016) and Sabater-Grande et al. (2023), who found decreased overconfidence by providing incentives for accurate postdictions of real exams their participants had taken. However, in both cases, better calibration was due to improved exam performance with incentives rather than decreased confidence judgments. Hence, most studies found a detrimental effect of incentives on the calibration of metacognitive confidence judgments, and resolution was only improved in the study by Lebreton et al. (2018). This effect was not replicated in the same paradigm by Hoven et al. (2022), however.

What is the state of the art for JOLs? We only found two studies incentivizing the accuracy of *predictive* metamemory judgments with money, namely Koriat et al., (2004, Experiment 6B) and Hasselhorn et al. (1987).^1^ Koriat et al. (2004) investigated whether accuracy incentives would reduce the neglect of the retention interval as a viable predictor of memory performance, but in their study, this had no effect: Although recall was much worse after a 1-week retention interval than a 10-min retention interval, incentivized JOLs did not reflect this difference. However, the incentives curiously reduced the overall JOLs as compared to a similar Experiment (6A) without monetary incentives, thereby contradicting the CJ boost effects reported above. Note, however, that a (non-randomized) comparison across experiments has to be interpreted with caution. Hasselhorn et al. (1987) did not elicit item-wise JOLs, but they asked for *aggregate* or *global* predictions of how many words from a list participants would freely recall. Participants first studied 12 lists of 32 words each (varying in difficulty operationalized by semantic categories and concreteness), and for each list predicted the number of words they would recall after a short retention interval. Half of the 80 participants were told that the six participants with the most accurate predictions would receive an additional amount of money. The listwise predictions were somewhat underconfident altogether (performance was better than the mean estimate), but the incentivized group was a bit more accurate in terms of calibration (15% vs. 22% underconfidence). Note that no measure of resolution was reported.

Contrary to postdictive CJs discussed above, the predictive judgments (item-by-item JOLs in Koriat et al. and aggregate predictions in Hasselhorn et al.) were rather *deflated* by monetary incentives, and potential effects on resolution were not reported. Before accepting this as an empirical fact, however, we want to discuss a serious methodological problem with incentivizing memory predictions that might have plagued both previous JOL studies and are particularly apparent in the studies by Callender et al. (2016) and Sabater-Grande et al. (2023) cited above.

### The challenge of incentivizing judgments of learning (JOLs)

In contrast to postdictions like CJs, predictions of one’s own behavior can be optimized via *two* possible routes: Either one can adjust the predictions to match the future behavior, or one can adjust the predicted behavior to match the predictions! In recent years, the discussion about JOL *reactivity* has highlighted that the criterion behavior is malleable and might be influenced by the very act of providing JOLs, either intentionally or unintentionally (Double & Birney, 2019; Mitchum et al., 2016; Soderstrom et al., 2015). If the experimenter grants a reward for matching predictions to actual behavior, one can of course also expect that participants with maximization interests may adjust their later behavior to maximize their reward. Consider the following thought experiment: A participant is told that they receive a reward for low JOLs accompanied by recall failure and for high JOLs accompanied by recall. This person might realistically think that they can safely recall six words from the list. Hence, they might just encode the first six words on the list and provide a JOL of 100% for each. During the remainder of the study phase, they ignore the presented study words while rehearsing the six first words, and they provide a 0% JOL for each ignored item. Of course, they will later recall only the six rehearsed words and leave the experiment with the maximum payoff. Replace “six” with any other number – the result of a perfect match would be the same. Hence, the participant could outsmart the experimenter with a simple trick, which would yield a severely distorted measure of JOL accuracy.

Also, less "wicked" strategies might be thought of: For example, the study and JOL phase are mastered in good faith, striving for maximum performance. However, during test, remembering having provided a low JOL during study might motivate to withhold a test response. In cued recall, remembering a high JOL associated with a cue word might motivate more effortful retrieval attempts, etc.

We do not claim that in Hasselhorn et al.’s (1987) or Koriat et al.’s (2004) study most participants used such strategies, but we cannot be sure. Even if only a few participants did so, this would counteract the goal to achieve veridical metamemory judgments and lead to artifactual apparent boosts of judgment accuracy in the aggregated data. At least, the theoretical rationale of economic research to provide a transparent optimization criterion is not fulfilled with a payoff scheme that can be “outsmarted” in such simple ways. Hence, a payoff scheme must incentivize *both* maximum memory performance *and* accurate memory predictions. Only if people try their best in the memory task *and* simultaneously receive rewards for accurate predictions can we be sure that they do not strategically adjust their memory performance.

**Fig. 1:**
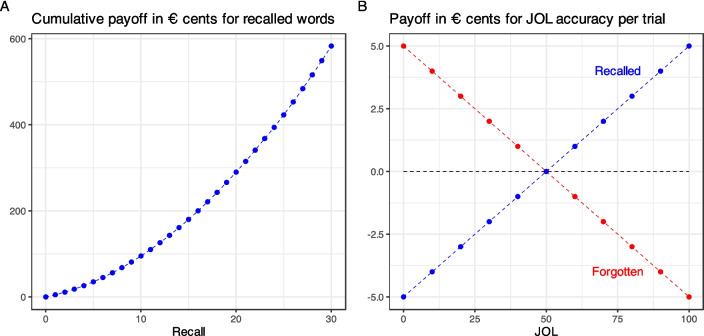
Payoff scheme in € cents used in the current experiments. The payoff for recall is positively accelerated (**A**). Payoffs for judgments of learning (JOL) accuracy follow a linear scheme with a maximum of additional 5 € cents reward or deduction for correct and incorrect responses, respectively (**B**)

In the experiments reported below, we used an incentive scheme that would always give a higher incentive to recalling as many words as possible by using the positively accelerated cumulative payoff function depicted in Fig. 1A. Participants were paid 5 cents for the first recalled word, an additional 6 cents for the second word, an additional 7 cents for the third word, and so on. Hence, recalling 15 words (of a list of 60) yields a payoff of 1.80 € and recalling 30 words would amount to 5.83 €. In addition, participants could receive in each trial a maximum payoff of 5 cents for accurate JOLs, as depicted in Fig. 1B. Extreme judgments yield 5 cents if correct (either a 0% JOL for a forgotten item or a 100% JOL for a recalled item) or a deduction of 5 cents if incorrect (0% for a recalled item, 100% for forgotten item). There is a linear payoff scheme for less extreme judgments with zero payoff for a non-informative 50% prediction. Note that with this payoff scheme, the benefit of recalling the next word (except the first one) will always exceed the potential reward or deduction due to the JOL prediction for this word, and, hence, maximum recall performance always pays off. In addition, however, a maximum of 3.00 € can be gained by optimal JOLs and a consistent linear scheme that encourages bold predictions and discourages non-informative predictions of 50%. These should only be used in case of pure guessing.

## Goal and research questions

Given the mediocre accuracy of JOLs reported in typical metamemory studies and the potential artifact risk of former JOL incentive experiments, our main goal was to assess whether there would be an increased accuracy motivation by proper incentivization that could potentially increase metacognitive accuracy. If incentivization had a positive effect, people’s metacognitive abilities might have been underestimated in the past. As Camerer and Hogarth (1999) pointed out in their review, the increased incentives might also backfire if they result in participants using more effortful but less accurate strategies (e.g., focus on the wrong cues for JOLs).

## Experiment 1

### Design

A between-subjects design with group as the sole factor (control group, JOL incentive group) was used. The control group was only incentivized for memory performance according to the function depicted in Fig. 1A. The JOL incentive group was additionally rewarded for accurate JOLs following the payoff function in Fig. 1B.^2^

### Materials

Sixty German four- to ten-letter nouns were selected from the 2,107 nouns from the BAWL-R database by Võ et al. (2009) according to the following procedure: To vary words broadly and orthogonally on the characteristics arousal, imagery, and frequency, words were first partitioned into four approximate quartiles of arousal ratings. Within each of these arousal categories, they were sorted into four imagery quartiles. Within these, they were sorted by frequency of usage and divided into quartiles again, resulting in 4 × 4 × 4 = 64 sets. From each set, one word was drawn in a way to ensure that mean values of arousal were similar across all levels of imagery and frequency (*M* = 2.99, *SD* = 0.01; rated on a 5-point scale, 1 = low arousal to 5 = high arousal), that mean values of imagery were similar across all levels of arousal and frequency (*M* = 4.16, *SD* = 0.01; rated on a 7-point scale, 1 = low imageability to 7 = high imageability), and that mean values of frequency were similar across all levels of arousal and imagery (*M* = 30.59, *SD* = 5.97; total frequency of appearance per million words). From the resulting list of 64 words, four low-frequency words were dropped.

### Procedure

The experiment consisted of a study phase with JOLs, a free recall test, and a personal significance assessment. Before the study phase, all participants were instructed to memorize 60 words for a later memory test, in which they would be asked to recall as many words as possible. They were also told that they would have to predict their chances of recalling each word in the test immediately after studying it. Afterward, they were informed that for every word they could recall during the test, they would be credited with a certain amount of money. They were told that for the first word recalled, they would receive 5 cents, for the second word recalled 6 additional cents, for the third word recalled 7 additional cents, and so on. They were explicitly told that recalling as many words at the test as possible would maximize the expected profit. They were shown the following examples: If you remember ten words, you will receive 5 + 6 + 7 + 8 + 9 + 10 + 11 + 12 + 13 + 14 = 95 cents, if you remember 15 words, you will get 5 + 6 + 7 + 8 + 9 + 10 + 11 + 12 + 13 + 14 + 15 + 16 + 17 + 18 + 19 = 180 cents, and if you recall all 60 words, you will get 20.70€. Participants in the control group were then presented with the question “*In order to maximize your winnings, it is important that in the test you…*”. The answer options were: “Only recall words to which you have assigned high probability judgments when learning them”; “Recall as many study words as possible”; and “Recall as few study words as possible.” If participants answered incorrectly, they were presented again with the explanation of the payoff scheme and the same question. This was repeated until the correct answer was provided.

Participants in the JOL incentive group were additionally informed that they could increase their reward by correctly estimating their recall probability by up to 5 cents per word, and that they could lose up to 5 cents per word by estimating incorrectly. They were told that the more extreme their probability rating is, the higher the profit or loss. Then, they were presented with a slider to set a specific recall probability and find out how many cents they could win or lose depending on whether they recall the word at the test or not. Participants were required to use the slider for at least five times and could continue using it until they felt that they had understood the scheme. Then, they were presented with the same question and procedure as the control group plus three more questions: (1) “*If you remember a word in the test, then …*”, answer options: “You lose money if your prediction is over 50%”; “You win additional money if the prediction is over 50%”; (2) “*If you do NOT remember a word in the test, then …*”, answer options: “You lose money if your probability rating is below 50%”; “You win additional money if the probability rating is below 50%”; and (3) “*For probability ratings that are extreme close to 0% or 100% …*”, answer options: “You can win a lot of money but not lose any money”; “You can win or lose a lot of money”; “You can lose a lot of money but not win any money.” If participants answered one or more questions incorrectly, the slider and the questions were presented again until all answers were correct. Before starting the study phase, participants in the JOL incentive group were reminded that even if their predictions were wrong, they could not lose more than the gain of remembering one word and that they could win up to 3 € extra with correct predictions.

At study, each word was presented at the center of the screen for 3 s preceded by a 500-ms fixation cross. Immediately after each word, the JOL prompt “Chance of recall (0–100%)?” appeared, and participants typed in any whole number from 0 to 100 (self-paced). A 100-ms blank screen preceded the next study trial. Following a 3-min distraction task consisting of easy abstract reasoning ability items (selecting one of four geometrical patterns that best completes a matrix) from Chierchia et al. (2019), participants had 5 min to type in as many studied words as they could remember. Then, they completed the personal significance assessment in which they were asked to indicate for each study word whether it had a special meaning for them personally (e.g., the word “strawberry” was particularly striking because strawberries are your favorite fruit or because you are allergic to strawberries) by clicking on buttons labeled “yes” and “no.”

### Participants

We aimed at *N* = 128 participants. This sample size provides a statistical power of (1—ß) = 0.80 to detect medium-sized main effects (*f* = 0.25, *d* = 0.50) with α = 0.05 in one-way ANOVAs and *t*-tests for independent samples (Faul et al., 2007). 128 participants were recruited at the University of Mannheim, most of them presumably students^3^ (*n* = 64 in the control group and *n* = 64 in the incentive group, 102 female, 24 male, two diverse). The mean age was 23.76 (*SD* = 6.46) years. Due to COVID-19, the experiment was conducted online with individual supervision via Zoom.^4^ To speed up data acquisition, the last 39 participants were offered 4 € on top of the money earned in the study by their performance and had the opportunity to donate the money to an organization of their choice rather than getting the money themselves. This change did not affect the results.

## Results

### Incentive effects on JOLs and memory performance

**Fig. 2:**
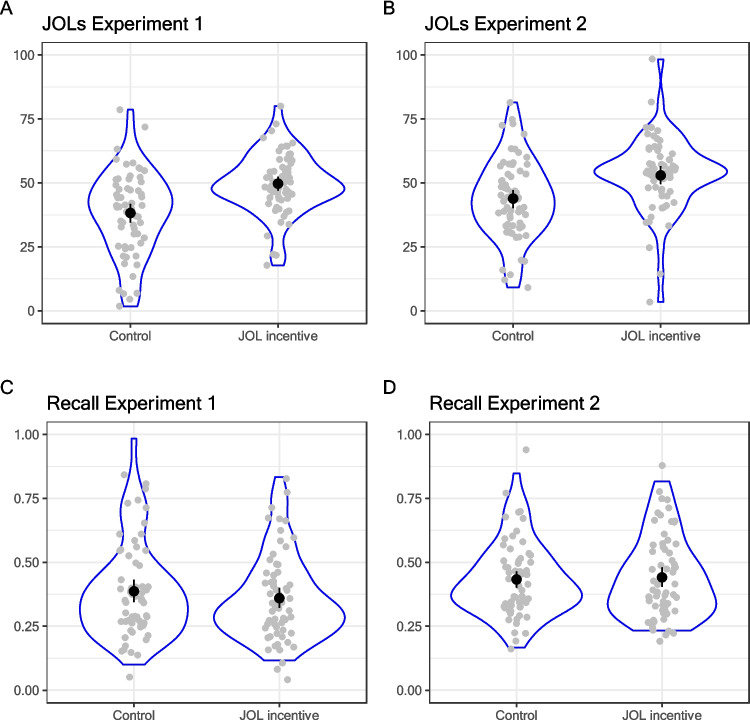
Mean judgments of learning (JOLs; top row) and mean recall performance (bottom row) in Experiments 1 (left column) and 2 (right column)

Panels A and C (left column) of Fig. 2 show mean JOLs and recall performance, respectively. The mean JOLs were higher in the incentivized group (*M* = 49.71, *SD* = 11.74) than in the control group (*M* = 38.2, *SD* = 16.03), *F*(1,126) = 21.47, *p* < 0.001, η_p_^2^ = 0.15. However, recall was not different across groups (*M* = 39% vs. 36% and *SD* = 18 vs. 16, respectively), *F*(1,126) = 0.79, *p* = 0.38, η_p_^2^ = 0.006.

### Incentive effects on JOL accuracy (resolution and calibration)

**Fig. 3:**
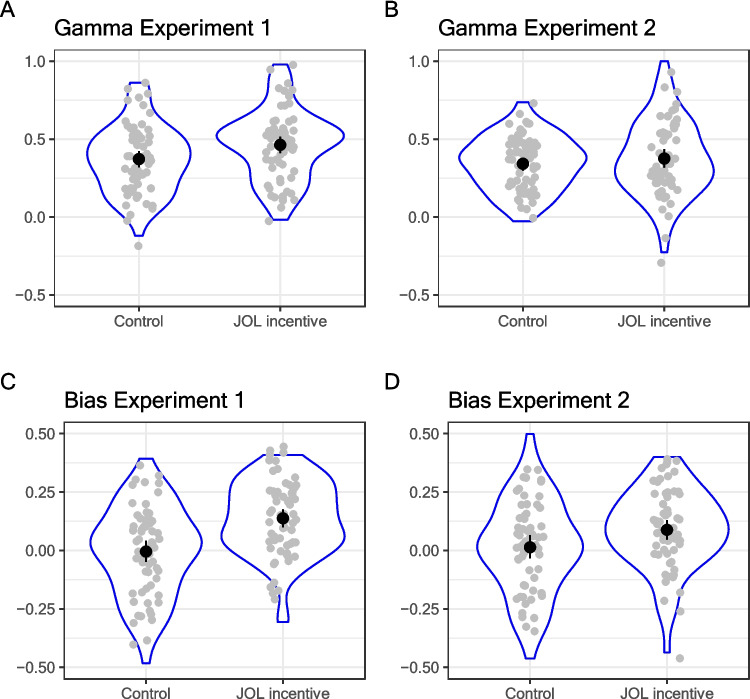
Metacognitive accuracy for Experiments 1 (left column) and 2 (right column), with resolution measured with Kruskal’s Gamma (top row) and bias (bottom row)

Kruskal’s Gamma was used as a measure of JOL resolution. Figure 3A shows that the mean Gamma values in the control condition (*M* = 0.35, *Md* = 0.37, *SD* = 0.27) were lower than in the JOL incentive condition (*M* = 0.46, *Md* = 0.5, *SD* = 0.22). The difference is of medium size and significant, *F*(1,126) = 6.58, *p* = 0.011, η_p_^2^ = 0.05, Cohen’s *d* = 0.46.^5^ This improvement in resolution was accompanied by pronounced average overconfidence in terms of mean bias: The difference between JOL (transformed to the same 0–1 scale as recall) and recall rate was on average positive in the incentive condition (*M* = 0.14, *SD* = 0.15, *t*(63) = 7.12, *p* = 0.001), while mean JOLs were unbiased in the control group (*M* = 0.005, *SD* = 0.19, *t*(63) = 0.2, *p* = 0.84; see Fig. 3C). Both groups differed significantly, *F*(1,126) = 21.74, *p* < 0.001, η_p_^2^ = 0.15, Cohen’s *d* = 0.83.

### Additional cue effects on JOLs and memory

Although not the main target of this research, we repeated the analysis of memory performance and JOLs including potential predictors at the item level. In particular, we included the personal significance of each word as indicated by the participant at the end of the study (see Undorf et al., 2022) as well as norming values of arousal, imagery, frequency, and the number of letters. We used a multilevel approach predicting the response to each item with these item-level predictors and the group difference as a participant-level predictor. We assumed random intercepts for participants. Recall was modeled with the *glmer* function in the *lme4* package (Bates et al., 2015) using a binomial link function,^6^ JOLs were analyzed as linear mixed models with the *lmer* function. We included the interaction of the item-level predictors with the group in the models. We only summarize the results here; the details can be found on the Open Science Framework at https://osf.io/s74m2/

When analyzing recall, there was still no effect of incentive group despite higher power (*z* = −0.42, *p* = 0.67) of the analysis. However, as one would expect, arousal, imagery, and personal significance significantly predicted recall of a word, all *z* > 2.06, all *p* < 0.04. For JOLs, we confirmed that average JOLs were higher in the incentivized group than in the control group, *t*(1199.86) = 2.64, *p* = 0.008. And again, arousal, imagery, and personal significance were significant predictors of JOLs, all *t*(> 7,500) > 4.4, all *p* < 0.001.

### Discussion

We found a medium-sized improvement in resolution due to incentivizing JOLs. However, JOLs were also boosted without an accompanying improvement of recall, producing marked overconfidence on average. The detrimental effect on bias was almost twice as large as the positive effect on resolution in terms of Cohen’s *d*. Also, according to the multilevel analysis using item level predictors and their non-significant interactions with the incentive group factor, incentives did not affect the cues people based their JOLs on.

## Experiment 2

Having shown a moderately beneficial effect of monetary incentives on resolution and a rather detrimental effect on calibration, Experiment 2 aimed at assessing the stability of these findings. A second motivation for Experiment 2 was the inclusion of an invalid cue in the set of predictors, which nevertheless affects JOLs. One such cue is *font size*, which has been shown to have a robust effect on JOLs (people assign higher JOLs to words written in large font than small font), whereas it does not affect memory, at least not to the same degree. Memory effects of font size are in most cases absent or tiny. The large discrepancy between JOLs and memory performance with respect to font size has been termed a “metacognitive illusion,” and it is a well-established phenomenon in the metamemory literature (see the meta-analysis by Chang & Brainerd, 2022; Rhodes & Castel, 2008; Undorf et al., 2018). One may dispute whether it should be termed an illusion when under certain boundary conditions, small memory effects are also observed, but the massive overestimation of the effect in JOLs justifies this term in our opinion. We consider this invalid cue an ideal testbed for probing the potentially beneficial effect of incentives. First, the salient font size is a strong demand cue that may drive participants’ JOLs through their attempt to please the experimenter or signal them that they picked up this feature in the procedure (Orne, 1962; see Mueller & Dunlosky, 2017, for a demonstration of a similar demand effect). Second, as cognitive misers, people might just go with the most salient cue. In both cases, incentives should reduce the illusion by encouraging participants to go with their true beliefs (rather than pleasing the experimenter) or think harder about truly predictive cues. If this works, the illusion should be reduced or even eliminated in an incentive condition.

### Design

The same basic design as in Experiment 1 was used.

### Materials

To homogenize word length, the second Experiment used 60 German four- to seven-letter nouns from Võ et al. (2009). To ensure a large variation and orthogonality of arousal and imagery values and keeping frequency approximately constant this time, the 2,017 words were first divided into arousal rating quartiles. Within those, imagery quartiles were built. From each of the 16 arousal-imagery combinations, four words were selected. These were assigned to the four font size conditions in a way to make sure that mean values of arousal were similar across all levels of imagery and font size (*M* = 2.77, *SD* = 0.01; rated on a 5-point scale, 1 = low arousal to 5 = high arousal), and that mean values of imagery were similar across all levels of arousal and font size (*M* = 4.38, *SD* = 0.04; rated on a 7-point scale, 1 = low imageability to 7 = high imageability). We also ensured that words in each level of arousal, imagery, and font size were similar in their frequency (*M* = 70.07, *SD* = 3.55; total frequency of appearance per million words). One noun from each font size level in the second and third levels of both arousal and imagery was dropped to obtain a study list of 60 nouns.

### Procedure

The same procedure as in Experiment 1 was used except for font size varying in four steps: From small to large, fonts were presented in 3.5%, 7%, 14%, and 25% of *viewport height*. Using this relative size unit ensured that the differences were retained regardless of the device and monitor participants used (tablet, laptop, desktop computer).

### Participants

Power analysis was identical to that of Experiment 1. All 129 participants were recruited at the University of Mannheim (*n* = 66 in the control group, and *n* = 63 in the incentive group, 85 female, 43 male, one provided no gender information). Mean age was 22.58 (*SD* = 3.58) years. Sixty-one participants completed the study online with supervision via Zoom, and 68 participants completed the study on PCs in the laboratory while being supervised. All participants were offered 4 € on top of the money earned by performance and the opportunity to donate the money (see Experiment 1).

### Results

#### Incentive effects on JOLs and memory performance

We report the results collapsed across both settings (lab and supervised Zoom sessions) since the setting had no main or interaction effects in any of the analyses. Panels B and D (right column) in Fig. 2 show mean JOLs and recall performance, respectively. Again, JOLs were on average considerably higher in the incentive condition (*M* = 52.92, *SD* = 14.43) than in the control condition (*M* = 43.83, *SD* = 15.24), *F*(1,127) = 12.06, *p* < 0.001, η_p_^2^ = 0.09. Also in line with Experiment 1, there was no effect of condition on recall (*M* = 0.44 vs. 0.43; *SD* = 0.15 vs. 14, respectively), *F*(1,127) = 0.10, *p* = 0.75, η_p_^2^ < 0.001.

#### Incentive effects on JOL accuracy (resolution and calibration)

Figure 3B shows that the mean Gamma in the control condition (*M* = 0.34, *Md* = 0.35, *SD* = 0.17) was slightly lower than in the JOL incentive condition (*M* = 0.38, *Md* = 0.35, *SD* = 0.24). The difference is numerically small and not significant, *F*(1,127) = 0.85, *p* = 0.36, η_p_^2^ = 0.007, Cohen’s *d* = 0.16. Again, the incentive produced overconfidence on average (*M* = 0.09, *SD* = 0.17, JOL scale transformed to the 0–1 interval), whereas the mean of JOLs was well calibrated in the control condition (*M* = 0.01, *SD* = 21), *F*(1,127) = 6.16, *p* = 0.014, η_p_^2^ = 0.046, Cohen’s *d* = 0.44. Hence, while the small improvement in resolution due to incentives from Experiment 1 was not replicated, incentives again produced significant overconfidence, which was almost three times as large in terms of Cohen’s *d* as the numerical improvement in resolution.

#### Additional cue effects on JOLs and memory

We analyzed whether JOLs and memory performance were predicted by the item predictors used in Experiment 1 (arousal, concreteness, frequency, number of letters, and personal significance) and font size. In the analysis of memory performance, only concreteness and personal significance reached significance (both *z* > 5.7, both *p* < 0.001). JOLs, in contrast, were also predicted by font size, *t*(7,187) = 4.31, *p* < 0.001, thus replicating the well-established font size metacognitive illusion (Chang & Brainerd, 2022; Rhodes & Castel, 2008; Undorf et al., 2018).^7^ Importantly, the font size/group interaction was not significant, *t*(7,187) = −1.27, p = 0.20, suggesting no significant reduction of the metacognitive illusion due to monetary incentives. Curiously, however, the difference in mean JOLs across groups found in the ANOVA was not significant in this analysis, *t*(2,645) = 1.22, *p* = 0.22.^8^

## Summary and general discussion

In two experiments, we incentivized participants for accurate item-wise predictions of their recall performance with a reward scheme that should prevent strategic adjustment of recall strategies by strictly encouraging maximum possible recall performance. Incentives had a small beneficial effect on JOL resolution in Experiment 1 which was not replicated in Experiment 2. A consistent result, however, was that rewarding JOL accuracy produced pronounced overconfidence at the group level in both experiments.

These results are thus in agreement with the studies of incentivizing postdictive confidence judgments cited above (Hoven et al., 2022; Lebreton et al., 2018, 2019; Meloy et al., 2006; Roch et al., 2011; Yates et al., 1997) that all reported an increase in overconfidence due to positive rewards. Only Lebreton et al. (2018) also reported an increase in the resolution of CJs, which was not replicated in the same paradigm by Hoven et al. (2022). Hence, these studies all agree in boosted confidence and only volatile resolution improvement. Our JOL results with proper incentivization are thus in line with the postdiction studies in which a strategic adjustment of the target behavior is not possible simply because it has already happened when the CJ is given.

Previous studies using *predictive* judgments, however, yielded results incompatible with ours by generally observing a *decrease* in JOLs due to incentivization (Callender et al., 2016; Koriat, 2004; Sabater-Grande et al., 2023) or a better calibration of predictions (Hasselhorn et al., 1987).

Why, then, do our results with predictive JOLs contradict the former studies with predictive judgments and more closely resemble those with postdictive CJs? Although this can only be a speculation at this point, we conjecture that our incentive scheme was effective in discouraging strategic memory adjustment by provoking maximum performance. In a similar vein, it does not make sense to adjust memory performance in a *postdictive* CJ task. Rather, the judgment is an assessment relative to the best possible performance. In contrast, all predictive metamemory tasks cited above entailed the possibility of adjusting later memory performance to the former predictions. Whereas there is no direct evidence reported for such a strategy in Hasselhorn et al. (1987) and Koriat et al. (2004), both Callender et al. (2016) and Sabater-Grande et al. (2023) report the improvement of exam performance due to incentives for accurate postdictions. In summary, this would mean that incentives cannot (or do not) improve metacognitive monitoring, but they can motivate participants to adjust their memory performance to the predictions. If this is either not possible (in postdictions) or not warranted (because of an incentive scheme discouraging memory adjustment), improvements will not be observed.

Why do positive incentives boost confidence? Worse calibration leads to a smaller payoff and hence works against the self-interest of participants. Meloy et al. (2006) point to the mediating role of positive mood indiscriminately inducing optimism. This interpretation is also favored by Lebreton et al. (2018) who invoke the "affect-as-information" framework (Schwarz & Clore, 1983) to explain why potential gains may boost confidence whereas potential losses dampen it. A decrease in overconfidence with negative mood states has been reported (Massoni, 2014) as well as a boost with positive mood states (Koellinger & Treffers, 2015). Hence, participants might factor in positive reward prospects into unwarranted high confidence.

Finally, one important result of Experiment 2 is the complete lack of an incentive effect on the font size metamemory illusion. Similar effect sizes of font size in the incentivized and the control condition in the absence of a corresponding memory effect underlines the stability of this illusion. In our view, this is a strong additional argument against an interpretation that would attribute the font size effect on JOLs as an expression of demand characteristics induced by the experimental setup (see Undorf et al., 2018).

Two potential limitations must be mentioned here: First, as one reviewer noted, the incentivization could change the *nature* of the JOL task in a way that does not only encourage participants to express their true probabilistic belief but transforms it into a betting task that elicits very different processes. For example, the hope that “post-decision wagering” would yield unbiased awareness measures has been questioned on the grounds that loss aversion is not adequately considered (Fleming & Dolan, 2010). If this objection were *generally* true, this would indeed seriously affect the validity of any incentivized judgment tasks (including JOLs). However, such a fundamental criticism would not only pertain to the current study but pose a problem for *all* studies using incentives for cognitive processing. Since incentivizing judgments is ubiquitous in the experimental economics literature, we would shy away from such a bold claim. We certainly do not deny the possibility of reactive effects of incentives on cognitive processes, but note that this is an empirical question and that the literature appears to have found mixed evidence in this regard, with most cited studies showing no reactive effects (see Charness et al., 2021, for a brief overview). Also, our general conclusion to remain rather skeptical of incentivizing JOLs to improve their accuracy is strengthened rather than weakened by this theoretical argument.

The second limitation may somewhat trouble experimental economists. Our incentive scheme follows a linear scoring rule (LSR) which is not strictly “proper” in the sense of theoretically maximizing the expected payoff when the stated probability (i.e., JOL) exactly matches one’s subjective belief (e.g., Murphy & Winkler, 1970). Rather, an LSR encourages more extreme responses. Theoretically proper incentive schemes are very complex through either using hard-to-understand penalty functions like quadratic or logarithmic scoring rules (Murphy & Winkler, 1970) or a series of choices between hypothetical lotteries for each judgment. While the latter is certainly not feasible for as many as 60 JOLs, the former are extremely hard to understand for participants and may also depend on their risk attitudes. Given that linear scoring rules have shown comparable outcomes to proper ones (Andersen et al., 2014; Charness et al., 2021), we employed the simpler rule here. Simplicity seemed particularly important in the context of the already complex memory incentivization that was required to motivate maximum performance. However, our conclusions are confined to situations like ours in which payoff schemes are adequate to incentivize higher resolution, but may not strictly conform to the theoretical optimum for eliciting numerically “true” beliefs.

## Conclusion

In two experiments, we found only inconclusive evidence for an improvement of JOL accuracy under conditions of proper incentivization for judgment accuracy in terms of resolution. What we found, however, is a consistent and indiscriminate boost of confidence leading to overconfidence, which is in line with many observations in the literature concerning confidence judgments. Also, the incentives did not reduce the impact of a metamemory illusion in Experiment 2. Regardless of the eventual explanation of the current results, the pragmatic implication for metamemory research is clear: Since the bias introduced is more pronounced than the potentially beneficial effect in terms of resolution, we advise against using incentives for JOL improvement.

## Data Availability

Raw data and analysis files to reproduce all analyses are available via the Open Science Framework (OFS) at https://osf.io/s74m2/

## Footnotes

1 Three other studies with incentivized JOLs are not reviewed here: Hacker et al. (2008) and Miller and Gerarci (2011) used additional course credits rather than monetary incentives for accurate predictions of exam results, and found no significant effects on calibration. In a study by Gutierrez and Schraw (2015), test performance rather than the metacognitive judgments was incentivized with money.

2 Readers may wonder why the control group got any incentives at all, but since our main focus was on the incentive effect on JOLs, the main comparison of interest of course must assure equal motivation for maximizing memory performance. Otherwise, different memory performance between groups might confound or mediate the incentive effect on JOLs to an unknown extent.

3 There is a small percentage of non-students in the participant pool of the university, but the vast majority of recruited participants are students. We did not ask for profession or study subject, though.

4 Note that the relatively high monetary stakes in this experiment might encourage cheating, which precluded an unsupervised online experiment in this case.

5 The results do not change when one extreme outlier with $\gamma$ = 1.0 in the control condition is removed, Cohen's *d* = .42.

6 To avoid convergence problems, the predictors were z-transformed, and the *nlminb* optimizing function was used.

7 In contrast to Experiment 1, the effect of arousal failed to reach conventional significance in predicting recall, *z* = 1.64, *p* = .10, or JOLs, *t*(7,187) = 1.86, *p* = .06.

8 The group effect is replicated, however, if all theoretically uninteresting interaction terms of item level predictors with group except font size are removed from the model, *t*(1,864) = 3.95, *p* < .001.

## Code availability

Code for analysis of data (R script) available on the OSF (see above).
